# Correlation of Dengue Warning Signs during Febrile Phase with Rotational Thromboelastometry, Cortisol and Feritin

**DOI:** 10.3390/ijerph19020807

**Published:** 2022-01-12

**Authors:** Syarifah Syahirah Syed Abas, Noralisa Abdul Karim, Petrick Periyasamy, Nurasyikin Yusof, Shamsul Azhar Shah, Tan Toh Leong, Saiful Safuan Md Sani, Hanita Othman, Sharifah Azura Salleh, Nurul Nadiah Mohd Zaidi, S Fadilah Abdul Wahid, Wan Fariza Wan Jamaludin

**Affiliations:** 1 Medical Department, Ampang Hospital, Ampang Jaya 68000, Selangor, Malaysia; sy_syahirah@yahoo.com; 2 Pusat Terapi Sel, Universiti Kebangsaan Malaysia Medical Centre, Cheras 56000, Federal Territory of Kuala Lumpur, Malaysia; noralisa@ppukm.ukm.edu.my (N.A.K.); nn.nurulnadiah@gmail.com (N.N.M.Z.); sfadilah@ppukm.ukm.edu.my (S.F.A.W.); 3 Infectious Disease Unit, Universiti Kebangsaan Malaysia Medical Centre, Cheras 56000, Federal Territory of Kuala Lumpur, Malaysia; petrick.periyasamy@gmail.com; 4 Hemostasis & Blood Transfusion Unit, Department of Diagnostic Laboratory Services, Universiti Kebangsaan Malaysia Medical Center, Cheras 56000, Federal Territory of Kuala Lumpur, Malaysia; syikinby@yahoo.co.uk; 5 Department of Community Health, Universiti Kebangsaan Malaysia Medical Center, Cheras 56000, Federal Territory of Kuala Lumpur, Malaysia; drsham@ppukm.ukm.edu.my; 6 Emergency Department, Universiti Kebangsaan Malaysia Medical Center, Cheras 56000, Federal Territory of Kuala Lumpur, Malaysia; sebastianttl@ppukm.ukm.edu.my; 7 Kuala Lumpur General Hospital, Jalan Pahang, Kuala Lumpur 50586, Federal Territory of Kuala Lumpur, Malaysia; dcicantab5@gmail.com; 8 Chemical Pathology Unit, Department of Diagnostic Laboratory Services, Universiti Kebangsaan Malaysia Medical Center, Cheras 56000, Federal Territory of Kuala Lumpur, Malaysia; drhanita@ppukm.ukm.edu.my; 9 Infection Control Unit, Department of Medical Microbiology and Immunology, Universiti Kebangsaan Malaysia Medical Center, Cheras 56000, Federal Territory of Kuala Lumpur, Malaysia; drazura@ppukm.ukm.edu.my

**Keywords:** cortisol, ferritin, dengue hemostasis, severe dengue, ROTEM, warning signs

## Abstract

Dengue mortality remains high despite monitoring against warning signs (WS). The associations of WS at febrile phase (FP) and hemorrhage at defervescence with the levels and kinetics of ROTEM, platelet count, cortisol, and ferritin were analyzed. Patients with confirmed dengue serology and WS in two centers were screened (*n* = 275) and 62 eligible patients were recruited prospectively over 9 months. “Vomiting” was the commonest WS (62.9%), with shortened clotting time (CT) INTEM (*p* = 0.01). “Hematocrit increase” showed significant prolonged CT INTEM, EXTEM, and FIBTEM (*p* < 0.05). “Platelet decrease” showed reduced platelet function and reduced clot amplitude at 10 min (A10) and maximum clot firmness (MCF) in INTEM and EXTEM (*p* < 0.001). The kinetics were reduced in platelet count, CT EXTEM, and cortisol (*p* < 0.05) but increased in CT INTEM (*p* = 0.03). At FP, “vomiting”, “hematocrit increase”, and “platelet decrease” demonstrated impaired CT, clot strengths A10/MCF and platelet functions. Majority (60/62, 96.7%) had non-severe outcomes, consistent with increase in cortisol kinetics. In conclusion, “vomiting”, “hematocrit increase” and “platelet decrease” at FP correlated with ROTEM. No conclusion could be made further regarding ferritin and cortisol. Larger study is required to study “hematocrit increase” with ROTEM as a potential marker for hemorrhage.

## 1. Introduction

Dengue mortality is a public health concern. Overall, 40% of world population live in dengue transmission areas [1]. From 2000 to 2014, dengue incidence has increased from 32:100,000 to 361:100,000 [2]. In 2017, the mortality rate was 0.21 compared to the World Health Organization (WHO) target of 0.14 [3]. Between January to March 2019, the cumulative case count rose to 157% higher than that of the same time points in the year 2018 [4].

Dengue fever runs through a dynamic and sequential phases of febrile, critical/defervescence, and recovery [2]. The WHO 1997 classification has clearly described three clinically distinct groups: (i) dengue fever, (ii) dengue hemorrhagic fever, and (iii) dengue shock syndrome [5]. However, there was a paucity of reference with regards to these dynamic phases to the clinical groups, and little applicability of the phase classification in dengue patients presenting organ failures without shock [6]. Similarly, up to 22% of dengue patients who presented with shock would not fulfil all four criteria for dengue hemorrhagic fever [7]. Hence, WHO addressed these issues in its 2009 classification, identified eight warning signs, and re-classified dengue severity into (i) dengue without warning signs, (ii) dengue with warning signs, and (iii) severe dengue [8]. These latest classifications improved triage criteria for admission and were more sensitive at detecting severe dengue [6]. However, concerns have arisen that the warning signs definitions were perhaps too broad and were largely based on expert opinions rather than well-designed clinical trials [7].

Conventional coagulation tests such as prothrombin time (PT), international normalized ratio (INR), and activated partial thromboplastin time (APTT) are primarily used clinically for anticoagulant drug monitoring. However, in dengue they are also used to monitor bleeding. More recently, rotational thromboelastometry (ROTEM), a useful bedside coagulation test, provides fast and clinically relevant information on clotting time, clot viscoelastic properties, and platelet function emulating in vivo hemostasis [9]. In dengue, thromboelastometry impairment, especially in INTEM (intrinsically activated) and EXTEM (extrinsically activated) pathways, were highly prevalent in patients with thrombocytopenia, despite normal conventional coagulations [10]. Thromboelastographic analysis in dengue also demonstrated predominant coagulation factor deficiencies, followed by low platelet functions [11].

It is generally known that sepsis and shock cause an elevated level in the stress hormone cortisol. In severe dengue, the serum cortisol level was found to be significantly higher than in other viral infections [12]. The cortisol level was also higher in severe dengue hemorrhagic fever than in dengue fever, suggesting its role as a possible biomarker for severity of dengue [13]. An acute phase protein ferritin and its potential as a biomarker to predict severe dengue was previously reported [14]. Dengue associated hemophagocytic syndrome, another severe complication of dengue fever, was also associated with hyperferritinaemia [15]. 

It is crucial to understand further the roles of warning signs in increasing triage accuracy and their impact on severe dengue. We conducted a prospective study to analyze the associations of warning signs during febrile phase with ROTEM. During defervescence, we analyzed the associations of hemorrhagic complications with ROTEM (INTEM, EXTEM, and FIBTEM (fibrin-based thromboelastometry test)), and the kinetics of ROTEM and biochemical markers such as ferritin and cortisol during progression from febrile phase into defervescence.

## 2. Materials and Methods

### 2.1. Recruitment

**Figure 1:**
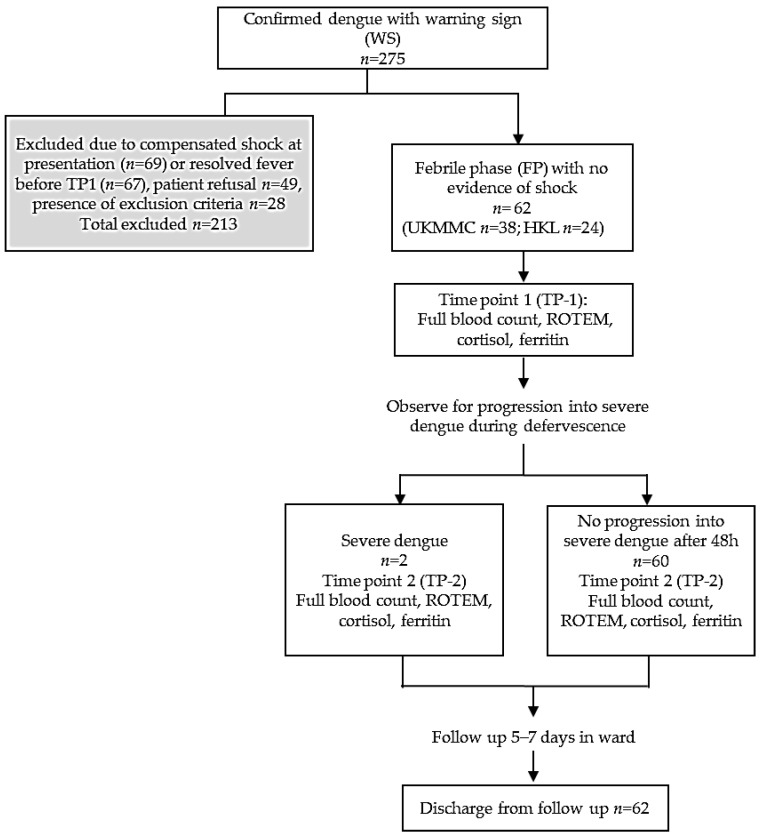
Study protocol and flow chart.

A prospective study was conducted in Universiti Kebangsaan Malaysia Medical Center (UKMMC) and Kuala Lumpur General Hospital (HKL) from June 2018–March 2019 (9 months). There were 275 patients aged ≥13-years with confirmed dengue and warning signs screened into the study, and 62 patients were eligible for recruitment (Figure 1). Dengue NS1 and IgM/IgG were performed using a rapid immune-chromatographic test (UKMMC) or SD Bioline^™^ Dengue Duo Rapid kit (Standard Diagnostics, Inc., Gyeonggi-do, Korea). The warning signs were defined as any of the following: abdominal pain, persistent vomiting >3/24 h, persistent diarrhea >3/24 h, third space fluid accumulation (ascites/pleural/pericardial effusion), mucosal bleeding, lethargy/restlessness/confusion, tender liver, and “hematocrit increase” with “platelet decrease” [8]. Median hematocrit values were 46% (male <60 years), 42% (male >60 years), and 40% (female all age) [2]. Platelet decrease was defined as ≤1.50 × 10^11^/L. The following were excluded: presence of compensated shock or resolved fever, active malignancy, HIV, autoimmune diseases, concomitant corticosteroids/chemotherapy, platelet inhibitors/antagonists, underlying hematological disorders, cirrhosis, or bone marrow metastasis. Time Point 1 (TP-1) referred to febrile phase while Time Point 2 (TP-2) referred to defervescence or onset of severe dengue. The study was approved by medical ethics committees (UKM FF-2018-126, NMRR-18-3124-44643 IIR) and all methods were performed in accordance with the relevant guidelines and regulations. Written informed consent were obtained from all participants and/or legal guardians.

### 2.2. TP-1

Dengue patients with warning signs in febrile phase were assessed upon recruitment for hemodynamic status/Grade 1 Common Terminology Criteria for Adverse Events (CTCAE) [16]. Blood samples were collected for platelet count, ROTEM analysis, PT/INR and APTT, ferritin and cortisol. Patients were admitted for observation and progression into defervescence.

### 2.3. TP-2

Patients in TP-1 were re-assessed upon recognition of severe dengue, or at 48 h later if there was no progression into severe dengue (TP-2) for hemodynamic, clinical, and hemorrhage complications/Grade ≥2 CTCAE [16]. Blood investigations were repeated as previously described. All patients were followed up in the ward for 5–7 days.

### 2.4. Hemostatic Parameters

Platelet count was extracted from full blood count data. Blood samples were drawn into citrate tubes and analyzed within 6 h using ROTEM (TEM International GmbH, Germany). The following data were measured and recorded: (i) clotting time from beginning of analysis until formation of 2 mm clot firmness (CT; seconds), (ii) clot amplitude at 10 min after CT (A10; mm), and (iii) maximum clot firmness (MCF; mm). The parameters CT, A10, and MCF real-time analysis were recorded for three coagulation pathways: (i) INTEM, (ii) EXTEM, and (iii) FIBTEM. INTEM assay is based on intrinsic activation by ellagic acid providing information on the coagulation factors involved in the intrinsic pathway. EXTEM assay uses tissue factor to initiate the extrinsic clotting cascade. FIBTEM assay measures the contribution of fibrin to clot strength which abolished the platelet contribution to clot formation by using a platelet inhibitor (cytochalasin D). Platelet functions were derived using formula subtracting MCF FIBTEM from MCF EXTEM (EXT_MCF_-FIB_MCF_). Blood samples from HKL were stored at 2–8 °C for <6 h before transported to UKMMC. Due to resource limitation, conventional coagulations (PT/INR and APTT) were performed for UKMMC subjects only (*n* = 38).

### 2.5. Biochemical Markers

Blood samples were drawn into BD vacutainer SST tubes. Blood samples from HKL were stored at 2–8 °C for <6 h before transported to UKMMC. Ferritin and cortisol were analyzed with immunoassay analyzer ARCHITECT i2000SR (Abbott Diagnostics, Lake Forest, IL, USA).

### 2.6. Clinical Assessments

Hemodynamic status and clinical signs were assessed at TP-1 and TP-2. Results were recorded according to CTCAE grading [16].

### 2.7. Statistical Analysis

Data were analyzed with SPSS Statistics software version 23. Categorical variables (gender, warning signs, signs of severe dengue) were presented as absolute frequencies. Continuous variables (weight, platelet count, ferritin, cortisol, conventional coagulations, ROTEM) were presented as median values with interquartile ranges (IQR). Mann–Whitney U tests were used for association between continuous variables with categorical data. Spearman’s rank correlation coefficients were used for correlation between hematocrit and platelet count with ROTEM. Wilcoxon Signed Rank was used for kinetics between variables in TP-1 and TP-2. The significance level was set at *p* < 0.05.

## 3. Results

### 3.1. Baseline Characteristics and Warning Signs at Febrile Phase (TP-1)

After screening, 213/275 dengue patients with warning signs at TP-1 were excluded due to presence of exclusion criteria, patient refusal, resolved fever or compensated shock. A total of 62 patients were recruited at TP-1 (Figure 1). Clinical characteristics, baseline platelet count and biochemical markers at TP-1 are shown (Table 1). The median age was 25.1 years (IQR 20–35.25) with 59.7% male. The median body mass index (BMI) was 24.45 kg/m^2^ (male) and 24.03 kg/m^2^ (female). Non-obese patients received intravenous fluid of 1.2–1.5 mL/kg/hour. Average time of fluid therapy before blood sampling at TP-1 was 2–8 h. Therefore, blood samples were taken when patients had received homogeneous rate of intravenous fluid. Majority presented during acute infection phase as reflected by NS1 antigen positivity (48/62, 77.4%) with median of Day 5 fever. The commonest warning sign was vomiting (39/62, 62.9%). Mucosal bleed occurred in 7 patients (11.3%) with gum bleeding (4/7) and epistaxis (3/7). The median platelet count was 9.65 × 10^10^/L, with hematocrit 43.2% (male) and 38.9% (female). ROTEM characteristics for the whole cohort and conventional coagulations for UKMMC subgroup (*n* = 38) are shown in Table 2. The APTT were prolonged in two patients who developed “mucosal bleed”.

### 3.2. TP-1: Association of Warning Signs at Febrile Phase with ROTEM and Platelet Function

At febrile phase, “vomiting” demonstrated significantly shorter clotting time in CT INTEM with 184 s (*p* = 0.01) compared to those with no vomiting (Table 3). For patients with “hematocrit increase with platelet decrease”, INTEM analysis showed significantly reduced clot amplitude A10 (*p* = 0.02) and reduced clot firmness MCF (*p* = 0.03). Similarly, in EXTEM analysis, “hematocrit increase with platelet decrease” showed reduced clot amplitude A10 (*p* = 0.01), reduced clot firmness MCF (*p* = 0.01) and significantly reduced platelet function (*p* = 0.02). We found no associations between any warning sign with FIBTEM abnormalities.

### 3.3. TP-1: Association of Warning Signs “Hematocrit Increase and Platelet Decrease” with ROTEM and Platelet Function

**Figure 2:**
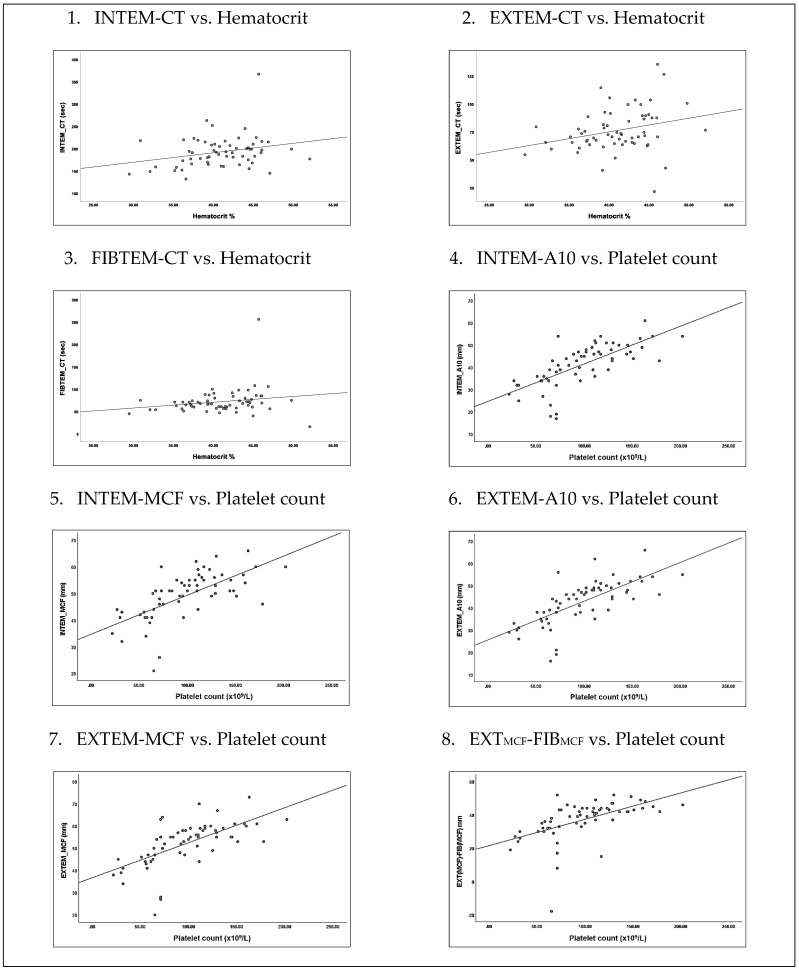
Association of “hematocrit increase” and “platelet decrease” during febrile phase with ROTEM and platelet function.

We analyzed this warning sign separately to see if “hematocrit increase” alone may have association with coagulopathy independent of low platelet factor (Table 4). “Hematocrit increase” correlated positively with all three ROTEM parameters, i.e., prolonged clotting time in CT INTEM (*p* = 0.04), EXTEM (*p* = 0.02), and FIBTEM (*p* = 0.04). In contrast, there were no correlation with platelet function. “Platelet decrease” correlated positively with reduced clot amplitudes and firmness across A10 INTEM (*p* < 0.001), MCF INTEM (*p* < 0.001), A10 EXTEM (*p* < 0.001), and MCF EXTEM (*p* < 0.001). Not surprisingly, “platelet decrease” correlated positively with reduced platelet function *p* < 0.001) and did not correlate with FIBTEM parameter (Figure 2).

### 3.4. TP-1 to TP-2: Kinetics of Hemostatic and Biochemical Markers between Febrile Phase and Defervescence

Following progression from febrile phase into severe dengue, or at 48 h later into defervescence phase, the hematocrit level increased significantly from 41.05% to 43.05% (*p* < 0.001) in keeping with expected increased capillary permeability and plasma leakage (Table 5). Here, platelet count also showed significant reduction from 9.650 × 10^10^/L to 6.550 × 10^10^/L (*p* < 0.001), consistent with current understanding of thrombocytopenia during defervescence phase. Clotting time kinetics were significantly increased in CT INTEM (192.50 s to 205 s, *p* = 0.03). In contrast, CT EXTEM was shortened from 73 s to 70 s (*p* = 0.01). In conventional coagulations for UKMMC subjects (*n* = 38), we detected shortening clotting times with PT 12.5 s from 13.4 s (*p* < 0.001), INR 0.95 from 1.03 (*p* < 0.001), and APTT 43.75 s from 48.85 s (*p* = 0.025). Cortisol showed down-going kinetics from 321.85 nmol/L to 254.35 nmol/L (*p* = 0.01). Ferritin demonstrated up-going kinetics from 2508.87 µg/L to 3765.37 µg/L but did not meet statistical significance.

### 3.5. TP-2: Association of Hemorrhage during Defervescence with ROTEM, Platelet Count & Conventional Coagulations

At severe dengue or 48 h into defervescence, hemorrhagic complications occurred in 17/62 with 16 non-severe gum bleeding (11/17), epistaxis (3/17), petechial rashes (2/17), and one severe intracerebral bleed. Hemorrhages were associated with reduced clot amplitudes and firmness at A10 EXTEM (*p* = 0.03), MCF EXTEM (*p* = 0.046), A10 FIBTEM (*p* = 0.049) and MCF FIBTEM (*p* = 0.04) (Table 6). In UKMMC subjects (*n* = 38), hemorrhages were associated with prolonged APTT (median 56.3 s, *p* = 0.01) (Table 7).

### 3.6. TP-2: Severity of Dengue

A total of 60/62 entered defervescence with non-severe dengue outcome. Only 2/62 progressed into severe dengue. The first severe patient was a 75-year-old female, who on Day 3 presented fever with persistent vomiting, lethargy, positive NS1, and negative IgM/IgG. Hematocrit was 35.1%, platelets were 129 × 10^9^/L, elevated cortisol was 610 nmol/L, and elevated ferritin was 40,000 µg/L. Conventional coagulations were normal. At Day 4, she developed severe pleural effusion. Her condition improved with supplemental oxygen and she discharged well on Day 8. The second patient was a 75-year-old female who on Day 5 presented fever with persistent diarrhea, lethargy, and positive NS1, IgM, and IgG. Hematocrit was 42%, platelets were 1.44 × 10^11^/L with elevated cortisol at 290 nmol/L and elevated ferritin at 2320 µg/L. APTT was mildly prolonged 45.5 s, but ROTEM was normal. On Day 6, she developed severe headache with GCS 8/15. CT brain revealed a right subdural bleed. At TP-2, APTT normalized 40.1 s, but ROTEM showed reduced platelet function and clot amplitude A10 INTEM. She succumbed on Day 13 due to complications from brain edema and cranial diabetes insipidus.

## 4. Discussion

Febrile phase was chosen as the baseline time-point (TP-1) to measure disease kinetics. A second time-point (TP-2) was identified as soon as severe dengue ensued, or at 48 h later after febrile phase as this was the well-established time interval of disease progression from febrile phase into defervescence [2]. 

Vomiting was the commonest reported warning sign in our cohort. Larger sample sizes will be needed to further understand its association with shortening of CT INTEM. Additionally, over-diagnosing vomiting as a warning sign could not be excluded as no further ascertainment could be made regarding history of accuracy, e.g., frequency of vomiting, retching, or amount of vomitus passed. 

The warning sign of “hematocrit increase and platelet decrease” as patients entered TP-2 from TP-1 were consistent with current knowledge in dengue pathophysiology and was frequently observed during capillary leakage in defervescence [2]. Here, this warning sign was also associated with reduced platelet function, clot amplitudes, and clot firmness in INTEM and EXTEM, but no abnormality in FIBTEM. When analyzed separately, “hematocrit increase” alone demonstrated ROTEM abnormalities with prolonged clotting time across INTEM, EXTEM, and FIBTEM, independent of thrombocytopenia. These may possibly be due to a global reduction in clotting factors concentration and fibrinogen defects consequent from capillary leakage [9,17]. In dengue, reduced Factor II, V, VII, VIII, IX, X, antithrombin, and α-2–antiplasmin were previously reported [18,19,20]. Predictably, we found no correlation between “hematocrit increase” alone with clot amplitudes and firmness. Conversely, “platelet decrease” correlated positively with reduced platelet function, clot amplitudes and firmness in INTEM and EXTEM. Under normal physiology, platelet adhesion and aggregation were required for primary hemostatic plug stability via interactions between von Willebrand factor (VWF) and platelets glycoproteins [21]. Clot strengths were predominantly contributed by platelets and, therefore, weakened in thrombocytopenia [22]. In dengue, impaired platelet aggregation to ADP from dengue antigen immune complexes formation on platelet surfaces have been reported [23,24]. A recent study analyzing the expression of endoglin (EDG) and Syndecan-1 (SDC1) from activated endothelial cells during plasma leakage in critical phase of dengue showed significant correlation in predicting dengue severity [25]. Hence, significant association of raised hematocrit with ROTEM abnormalities as found in this study has further potential for translational research in the future. 

The increasing kinetics in hematocrit and reducing kinetics in platelet level observed between TP-1 and TP-2 were consistent with current knowledge and understanding of dengue infection. The clotting time kinetics in INTEM were significantly increased. The number of bleeding episodes also increased from 7 patients at TP-1 to 17 patients at TP-2. The observed increased in INTEM kinetics at TP-2 with increased in bleeding tendencies may partly be explained by hepatocyte injury during defervescence that affected clotting factors production, especially in the intrinsic pathway [26,27]. Out of 17 patients with hemorrhagic complications, 11 had liver functions tests taken and 9 were deranged with elevated ALT more than 5× upper limit, ranging between 250 to 750 IU/L (82%). In contrast, the clotting time kinetics in EXTEM were significantly reduced and similar pattern of reduction was observed in PT/INR and APTT, despite an increased in bleeding episodes at TP-2. This may partly be explained activation of extrinsic pathway by tissues factors from endothelial cell injury and leaked capillary, or an increase in VWF, a known acute phase reactant [28]. Hence, the kinetics and pattern of EXTEM and conventional coagulation tests between febrile phase and defervescence may have no clinical significance for monitoring against bleeding. However, this study was not focused nor powered to address this issue. Meanwhile, cortisol showed reduced kinetics consistent with non-severe outcomes observed in our cohort. Higher cortisol with mean levels of 655.4–844.6 nmol/L have been reported in severe dengue [12,13]. Corticosteroids may benefit children >8 years, but subsequent studies failed to demonstrate its superiority [29,30,31]. However, corticosteroids are still recommended in severe dengue complicated by hemophagocytosis, myelitis, or encephalomyelitis [2,32]. Ferritin association with dengue severity have been reported [14,15,33]. Here, ferritin showed increasing kinetics at TP-2, and this was probably consistent with its function as an acute reactant protein. However, the kinetics were not significant statistically, possibly due to the small number of severe dengue outcomes in our cohort.

At TP-2 defervescence, the bleeding episodes were significantly associated with reduced clot amplitudes and firmness in both EXTEM and FIBTEM, in parallel with decreased kinetics in platelet count, suggestive of qualitative defect in fibrinogen activities. Amongst UKMMC subjects (*n* = 38), 11/38 hemorrhages at TP-2 were significantly associated with prolonged APTT, but no significant association with INTEM at this point. No further interpretation could be made in view of small sample size.

Only 2/62 developed severe dengue outcomes which may reflect effective in-patient dengue monitoring. Age >65-years deserve extra precautions as advanced age is criteria for hospital admission [2]. However, the use of warning signs as triage criteria in highly endemic regions may cause unnecessary admissions and resource allocation [6,34]. The rate of secondary dengue with IgG positivity previously associated with severe dengue was low in our study (3.2%) [35,36]. Only one mortality was recorded after defervescence phase (*n* = 1/62) in a 75-year-old female patient with persistent diarrhea with no vomiting. Unfortunately, we did not find significant association between diarrhea and ROTEM parameters. In this case, ROTEM analysis at defervescence and at development of right subdural bleeding demonstrated reduced platelet function and reduced clot amplitude at INTEM A10. Overall, we found no significant association between hemorrhage and INTEM A10. The possible mechanisms given abnormal finding of INTEM A10 in this mortality should be explored in future study with a larger number of severe dengue outcomes.

## 5. Conclusions

“Mucosal bleed” at febrile phase was not found to be associated with ROTEM parameters or PT/INR. Extrinsic pathways may not be reflective of bleeding manifestations during febrile phase. However, cautious interpretation is required given our small sample size.

The association between “hematocrit increase” with prolonged clotting time across all ROTEM parameters deserves further analysis with larger sample size to elicit its potential predictive role as a biomarker for hemorrhage.

The reduction in PT/INR and APTT kinetics between febrile phase and defervescence despite an increase in hemorrhagic episodes may indicate that conventional coagulation tests were not reflective of bleeding complications especially if they were normal at febrile phase. At defervescence, VWF may have helped APTT recovery during defervescence. The association between hemorrhage with reduced ROTEM clot amplitude and firmness at defervescence and the association between transaminitis with ROTEM also warrant further assessment size.

The reduction in cortisol kinetics was consistent with non-severe dengue outcomes. Although ferritin demonstrated increased kinetics, it was not statistically significant. The roles of cortisol and ferritin as potential biomarkers for dengue need to be explored further in a larger study.

## Figures and Tables

**Table 1:** Clinical characteristics, platelet indices and biochemical markers of dengue patients with warning signs in febrile phase at TP-1 (*n* = 62).

Characteristic	Median (IQR Q1–Q3) or *n*/62 (%)
Age, years	25.1 (20–35.25)
Male	23 (19–32)
Female	29 (22–45.5)
Gender	
male	37 (59.7%)
female	25 (40.3%)
BMI (kg/m^2^)	
Male	24.45 (21.59–28.78)
Female	24.03 (20.68–27.54)
Day of fever	5 (4–5)
Confirmed dengue fever	
NS 1 positivity	48 (77.4%)
IgM positivity	8 (12.9%)
IgG positivity	2 (3.2%)
IgM & IgG positivity	4 (6.5%)
Warning sign present	
Abdominal pain	34 (54.8%)
Vomiting >3/24 h	39 (62.9%)
Loose stool >3/24 h	28 (45.2%)
Mucosal bleeding	7 (11.3%)
Lethargy, confusion	26 (41.9%)
Tender liver	0
Clinical fluid accumulations	0
HCT increase with platelet decrease	27 (44%)
Blood parameters during febrile phase	
Platelet count (×10^9^/L)	96.5 (65–125.75)
Mean platelet volume, MPV (fL)	11.06 (10.45–11.73)
Plateletcrit (%)	0.11 (0.08–0.13)
Hematocrit %	
Male	43.2 (40.05–45.2)
Female	38.9 (35.65–41.05)
Cortisol (nmol/L)	321.85 (256.68–409.35)
Ferritin (µg/L)	2508.87 (798.30–5217.25)

**Table 2:** Hemostasis characteristics of dengue patients with warning signs febrile phase at TP-1.

ROTEM Parameters (*n* = 62)	Median (IQR Q1–Q3)	Laboratory Normal Range
INTEM		
CT (s)	192.5 (170.5–211)	100–240
A10 (mm)	43 (34.75–48)	44–66
MCF (mm)	51 (43.75–55)	50–72
EXTEM		
CT (s)	73 (65.75–88.25)	38–79
A10 (mm)	44 (36.5–49)	43–65
MCF (mm)	55 (46.75–59)	50–72
FIBTEM		
CT (s)	69 (60.5–81)	n. d
A10 (mm)	13 (10–15.25)	7–23
MCF (mm)	14 (11–17)	8–24
EXT_MCF_-FIB_MCF_ (mm)	40.5 (32–44)	41–48
Conventional coagulation tests (*n* = 38)		
INR	1.030 (0.98–1.10)	0.8–1.1
PT (s)	13.4 (12.8–14.32)	11.8–14.5
APTT (s)	48.85 (43.68–52.28) *	30–44.5
Fibrinogen (g/L)	2.7 (2.28–3.0)	2–4

* At TP-1, our patients have prolonged APTT with median of 48.85 (normal range 30–44.5).

**Table 3:** Association of warning signs in febrile phase with ROTEM (TP-1).

Warning Signs	INTEM Median (IQR)			EXTEM Median (IQR)			FIBTEM Median (IQR)			Platelet Function (EXT_MCF_-FIB_MCF_) Median (IQR)
*n* = 62	CT (s)	A10 (mm)	MCF (mm)	CT (s)	A10 (mm)	MCF (mm)	CT (s)	A10 (mm)	MCF (mm)	
Abdominal pain										
Yes, *n* = 34	192 (168.25–217.5)	43.5 (35.5–48.5)	51 (45.5–56.25)	72 (64–90)	45 (38–55)	55 (47.75–59)	68.5 (58.75–81)	13 (10–15)	14.5 (11–16.25)	41.5 (33–44.25)
No, *n* = 28	194.5 (176–205)	43 (34–46.75)	50 (41.5–53.75)	73.5 (68–87.75)	44 (35–48)	53.5(44–58)	69.5 (61.25–83.25)	13 (9.25–16)	14 (10.25–17.75)	37 (29.75–43)
*p* value	0.72	0.53	0.24	0.63	0.41	0.25	0.99	0.84	0.98	0.17
Vomiting >3/24 h										
Yes, *n* = 39	184 (165–200) *	43 (36–49)	50 (43–55)	73 (67–88)	44 (38–50)	53 (47–59)	69 (62–75)	13(10–16)	14(11–17)	41 (33–44)
No, *n* = 23	203 (184–224)	44 (32–47)	51 (44–55)	73 (57–93)	44 (33–49)	55 (45–58)	73 (57–93)	13 (10–15)	15 (11–16)	40 (30–44)
*p* value	0.01	0.43	0.93	0.64	0.71	0.74	0.73	0.74	0.68	0.94
Diarrhea >3/24 h										
Yes, *n* = 28	200 (179.75–214.75)	43.5 (34–46.75)	51 (42.25–55)	73.5 (65.5–84)	45 (34.25–48)	55 (44.25–58)	70.5 (58–77.5)	12.5 (10–15.75)	13.5 (11–17.5)	40 (30–44)
No, *n* = 34	184 (165.75–205)	43 (36–49.25)	50.5 (45.5–57)	72 (65.75–89.25)	44 (38–50.25)	54 (47.75–59)	67.5 (60.5–82.25)	13 (10–15.25)	14.5 (11–17)	40.5(34.5–44)
*p* value	0.18	0.51	0.48	0.85	0.51	0.83	1	0.99	0.95	0.5
Mucosal bleeding										
Yes, *n* = 7	201 (156–221)	37 (28–49)	47 (35–60)	73 (61–81)	37 (29–52)	48 (38–60)	68 (52–73)	12 (8–15)	13 (7–19)	37 (19–44)
No, *n* = 55	192(171–211)	44 (35–48)	51 (44–55)	73 (66–89)	45 (38–49)	55 (47–49)	69 (61–81)	13 (10–16)	14 (11–17)	41 (32–44)
*p* value	0.87	0.35	0.77	0.67	0.3	0.27	0.68	0.48	0.55	0.5
Lethargy, confusion										
Yes, *n* = 26	200 (173.25–20.5)	43 (34–48.25)	50.5 (41–55)	74.5 (66–90.25)	44 (34–49.5)	54.5 (43.75–59)	70 (63.5–79.75)	12.5 (10–15.25)	13.5 (11–17.25)	39 (31.5–43.25)
No, *n* = 36	192 (169.5–218.75)	44 (36–47.75)	51 (46–55.75)	72 (64.25–81.5)	45 (38–48.75)	55 (48.25–58.75)	68.5 (58–81)	13(10–15.75)	14.5 (11–16.75)	41 (33.5–44)
*p* value	0.88	0.53	0.27	0.26	0.66	0.42	0.75	0.87	0.99	0.46
“HCT increase” with “platelet decrease”										
Yes, *n* = 27	200 (180–208)	41 (34–46) **	49 (41–53) **	73 (66–90)	42 (33–47) **	52 (44–56) **	70 (58–85)	13 (10–15)	14 (11–17)	38 (30–42) **
No, *n* = 35	184 (165–216)	45 (38–51)	51 (47–57)	73 (65–82)	46 (39–52)	55 (50–61)	68 (61–76)	13 (10–16)	14 (11–17)	42 (35–46)
*p* value	0.19	0.02	0.03	0.64	0.01	0.01	0.89	0.94	0.98	0.02

* Significantly reduced as compared to those without vomiting; ** Significantly reduced as compared to those without “HCT increase” with “platelet decrease”. Statistical analysis used was Mann–Whitney U test.

**Table 4:** Association of “hematocrit increase” and “platelet decrease” during febrile phase with ROTEM and platelet function.

Parameters	Hematocrit Level, Spearman’s Rho (*p*-Value)	Platelet Count, Spearman’s Rho (*p*-Value)
INTEM		
CT (s)	0.26 (*p* = 0.04) *	−0.23 (*p* = 0.07)
A10 (mm)	−0.21 (*p* = 0.10)	0.77 (*p* < 0.001) *
MCF (mm)	−0.20 (*p* = 0.11)	0.71 (*p* < 0.001) *
EXTEM		
CT (s)	0.31 (*p* = 0.02) *	−0.06 (*p* = 0.62)
A10 (mm)	−0.19 (*p* = 0.15)	0.78 (*p* < 0.001) *
MCF (mm)	−0.22 (*p* = 0.09)	0.71 (*p* < 0.001) *
FIBTEM		
CT (s)	0.27 (*p* = 0.04) *	−0.06 (*p* = 0.62)
A10 (mm)	−0.21 (*p* = 0.11)	0.11 (*p* = 0.40)
MCF (mm)	−0.22 (*p* = 0.09)	0.06 (*p* = 0.65)
EXT_MCF_-FIB_MCF_ (mm)	−0.1 (*p* = 0.44)	0.70 (*p* < 0.001) *

* Significant *p*-value < 0.05. Association of these parameters were tested using Spearman’s rank correlation coefficient (*n* = 62).

**Table 5:** The kinetics of hemostatic and biochemical markers in dengue with warning signs between febrile phase and defervescence.

Parameters *n* = 62	Febrile Phase, TP-1 Median (IQR)	Defervescence, TP-2 Median (IQR)	*p*-Value	Laboratory Normal Range
Hematocrit (%)	41.05 (37.88–44.33)	43.05 (38.98–46.45) *	<0.001	38–46
Platelet (10^9^/L)	96.50 (65–125.8)	65.50 (37.5–104.25) *	<0.001	150–400
INTEM				
CT (s)	192.50 (170.5–211)	205 (178.5–224) *	0.03	100–240
A10 (mm)	43 (34.75–48)	40.5 (33–48.25)	0.11	44–66
MCF (mm)	51 (43.75–55)	50.0 (43–59)	0.99	50–72
EXTEM				
CT (s)	73 (65.75–88.25)	70 (63.75–75.25) *	0.01	38–79
A10 (mm)	44 (36.5–49)	40.28 (32.75–51)	0.08	43–65
MCF (mm)	55 (46.75–59)	52.18 (44–61.25)	0.74	50–72
FIBTEM				
CT (s)	69 (60.5–81)	67.63 (59.75–73.25)	0.27	n. d
A10 (mm)	13 (10–15.25)	13.02 (10–16)	0.79	7–23
MCF (mm)	14 (11–17)	15.68 (12–18.25)	0.27	8–24
EXT_MCF_-FIB_MCF_ (mm)	40.5 (32–44)	36.75 (31.75–42)	0.3	41–48
Conventional coagulation, (UKMMC subjects *n* = 38)				
INR	1.03 (0.98–1.10)	0.95 (0.92–0.98) *	<0.001	0.8–1.1
PT (s)	13.4 (12.78–14.35)	12.5 (12.18–12.9) *	<0.001	11.8–14.5 s
APTT (s)	48.85 (43.55–55.3)	43.75 (39.2–54.35) *	0.025	30–44.5 s
Fibrinogen g/L	2.7 (2.28–3.0)	2.75 (2.5–3.1)	0.18	2–4 g/L
Cortisol (nmol/L)	321.85 (256.68–409.35)	254.35 (202.98–366.25) *	0.01	>50
Ferritin (µg/L)	2508.87 (798.30–5217.25)	3765.37 (1431.83–6393.40)	0.06	4.63–204

* Significantly different as compared to febrile phase (*p*-value < 0.05). Statistical analysis used was Wilcoxon Signed Rank (*n* = 62).

**Table 6:** Association of hemorrhage during defervescence with ROTEM and platelet count.

	INTEM Median (IQR)			EXTEM Median (IQR)			FIBTEM Median (IQR)			Platelet Function (mm) (EXT_MCF_-FIB_MCF_)	Platelet Count (10^9^/L)
	CT (s)	A10 (mm)	MCF (mm)	CT (s)	A10 (mm)	MCF (mm)	CT (s)	A10 (mm)	MCF (mm)		
No hemorrhage, *n* = 45	205 (174–223)	41 (37–48)	51 (45.5–60)	70.6 (64–76)	41 (37–48)	55 (47.5–62)	67.63 (59–72)	14 (12–16)	15.68 (13–19)	39 (32.5–43)	76.5 (36.35–124)
Hemorrhage *n* = 17	207 (193–236.5)	36 (21–45)	46 (29.5–56.5)	69.5 (64–74)	35.5 * (25.5–42)	47 * (34–67.3)	66.5 (69–76)	10.5 * (8–15)	12 * (10–19.6)	34.5 (22–42)	46 (20–82)
*p* value	0.44	0.18	0.18	0.41	0.03	0.046	0.61	0.049	0.04	0.34	0.13

* Significantly decreased as compared to “No hemorrhage” (*p*-value < 0.05). Statistical analysis used was Mann–Whitney U test (*n* = 62).

**Table 7:** Association of hemorrhage during defervescence with conventional coagulation profile (UKMMC subjects, *n* = 38).

	INR Median (IQR)	PT (sec) Median (IQR)	APTT (sec) Median (IQR)	Fibrinogen g/L Median (IQR)
No hemorrhage *n* = 27	0.94 (0.88–0.98)	12.5 (11.9–13.05)	43.3 (38.9–49.8)	2.85 (2.5–3.18)
Hemorrhage *n* = 11	0.95 (0.92–1.0)	12.6 (12.3–13.1)	56.3 (42.1–64.9) *	2.6 (2.5–3)
*p* value	0.17	0.12	0.01	0.43

* Significantly increased as compared to “No hemorrhage” (*p*-value < 0.05). Statistical analysis used was Mann–Whitney U test (*n* = 38).

## Data Availability

The data presented in this study are available on request from the corresponding author.
